# Impact of the COVID-19 health crisis on psychotropic drug use in children and adolescents in France

**DOI:** 10.1186/s13034-024-00806-z

**Published:** 2024-09-16

**Authors:** Jérémy Couturas, Jérémy Jost, Laurence Schadler, Nicolas Bodeau, Véronique Moysan, Bruno Lescarret, Bertrand Olliac, Benjamin Calvet

**Affiliations:** 1 Pharmacy Departement, Centre Hospitalier Esquirol, 87000 Limoges, France; 2 Inserm U1094, IRD UMR270, Univ. Limoges, CHU Limoges, EpiMact- Epidemiology of chronic diseases in tropical zone, Institute of Epidemiology and Tropical Neurology, OmegaHealth, Limoges, France; 3 Centre Memoire de Ressources Et de Recherche du Limousin, Centre Hospitalier Esquirol, 87000 Limoges, France; 4 University center for adult and geriatric psychiatry and addictology, Centre Hospitalier Esquirol, 87000 Limoges, France; 5 Clinical Pharmacy Unit, Pharmacy Department, University Hospital of Limoges, Avenue Martin Luther King, 87000 Limoges, France; 6 Child and Adolescent Psychiatry Faculty of Medicine, 2 rue du docteur Marcland, 87025 Limoges, France; 7 French National Health Fund, Bordeaux, France; 8 Centre Hospitalier Esquirol, Research and Innovation unit, 87000 Limoges, France; 9 Departement of child and Adolescent Psychiatry, Centre Hospitalier Esquirol, Limoges, France

**Keywords:** Mental health, Child psychiatry, Adolescent psychiatry, COVID-19, Psychotropic drugs, Health insurance

## Abstract

**Background:**

In 2019, the world faced a pandemic brought about by a severe acute respiratory infection caused by SARS-CoV-2 virus. The spread of this virus has profoundly affected societies, particularly in terms of their economic, human and social dimensions, as well as their healthcare systems. Several restrictive measures (reduced social interaction, periodic school closures,…) had to be taken to contain the spread of the virus. These measures have had an impact on the psychological well-being of both adults and children. The aim of this study was to assess the changes in psychotropic drugs prescriptions for children and adolescents living in Limousin, a French region, over the period 2018 to 2021.

**Methods:**

The consumption of psychotropic drugs was studied using a national database of drug reimbursement. These data were extracted and supplied from the nationwide French reimbursement healthcare system database (SNDS). The following therapeutic classes were studied: N05A (antipsychotics), N05B (anxiolytics), N05C (hypnotics and sedatives), N06A (antidepressants) and N06B (psychostimulants). Data were collected for insured persons under the age of 18 who received at least one reimbursement for a psychotropic drug between 2018 and 2021.

**Results:**

Over a 4-year period, 7949 patients under the age of 18 were included with an average age of 12.1 years and a sex ratio of 0.97 M/F. The number of patients increased from 2018 to 2021, as did the number of reimbursements. We observed a statistically significant difference of means of patients reimbursed per week for on five therapeutic classes, with the greatest difference in 2021 (p < 0.0001). An increase in the number of patients of between + 20.7% and + 689% was observed, depending on the drug classes studied. Comparisons between the COVID-19 and pre-COVID-19 periods showed a significantly higher COVID average for psychotropic drugs reimbursements in general and individually for all classes except psychostimulants.

**Conclusion:**

The results show a significant increase in the consumption of psychotropic drugs among youth. The increase in psychotropic drug use was continuous and progressive throughout the pandemic. All five classes were increased, but particularly anxiolytics and antidepressants. The COVID-19 context may have been at the origin of a deterioration in the mental health of children and adolescents, or of a heightened awareness of psychiatric care among young people.

**Supplementary Information:**

The online version contains supplementary material available at 10.1186/s13034-024-00806-z.

## Introduction

In the last quarter of 2019, the emergence of a new virus, SARSCoV-2, triggering a pathology in humans known as COVID-19 (COronaVIrus-Disease 2019), led to a pandemic [1]. The high transmission capacity of this virus and the lack of effective therapy contributed to its rapid spread, officially reaching France in early 2020 [2]. The disease has led to the setting of restrictive, unprecedented and obligatory national actions in an attempt to contain its spread and reduce its consequences in terms of both public health and the economy [3, 4].

These measures, restriction of movement and confinement of population, reduction of social interactions, and periodic school closures, have had an impact on the psychological well-being of people.

A national study conducted by the French drug agency has highlighted the increase in the use of psychotropic drugs in 2020 and early 2021 in the general population. The rise in the use of these drugs seems to indicate a deterioration in the mental health of the French population in the face of the health crisis [5]. In France, prior to the COVID-19 crisis, it was estimated that 1% to 2% of prepubescent children suffer from characterized depressive episodes, and up to 8% of adolescents [6–8]. The prevalence of bipolar disorder among adolescents is estimated at 1.8% [9]. In addition, 4.6% of 7–11-year-olds and 3.3% to 7.3% of adolescents are thought to suffer from anxiety disorders [10].

In March 2020, the first measures taken by the health authorities consisted of the timely closure of schools in geographical areas of France where active clusters were present. Next, the interruption of activities in nurseries, schools and universities was implemented. Friends and family gatherings were no longer permitted. Distance learning has been introduced in the most affected departments. Schools were reopened and were marked by the implementation of the health protocol with the obligatory wearing of masks from the age of 11[11]. Following, several measures were gradually introduced as self-test at school, voluntary vaccination for 12–17-year-old pupils, and eventually vaccination for children aged 5 to 11 considered to be at risk [12]. In France, during 2020 there were two containments (spring and autumn) and in 2021 one containment (spring) with a first half year on curfew.

During previous epidemics such as the avian flu or H1N1 in 2009, or SARS in 2003, mental disorders have been observed in children. A retrospective study has shown that about 17.0% of children suffer from adjustment disorders, stress and bereavement. The impact of these events resulted in up to 30.0% suffering from post-traumatic stress disorder for those who had been isolated and/or confined [13, 14]. A review of the after-effects of previous epidemics (H1N1, H5N1, SARS, etc.) revealed a negative impact of quarantine or social isolation. It revealed the appearance in children of symptoms such as anxiety, fear or post-traumatic stress, etc. [15].

From the beginning of the pandemic, negative effects were reported [16]. Some studies showed that the quality of life of children and adolescents deteriorated with a change in behavior such as weight gain in 41.5% of minors [17], sleep disorders in 21.0% of children and adolescents [18], an increase in internet use in 63.3% [17], irritability in 31.0% of minors and attachment problems with parents in 36.0% [18].

A Chinese study [19] questioned children and adolescents aged 12 to 18 during the first wave of the epidemic. A proportion of 43.7% of them reported depressive symptoms, 37.4% anxiety and 31.3% both. In addition, this work highlighted a greater prevalence of depressive and anxiety symptoms in adolescents. A second study [20] confirmed this result with a higher level of anxiety, social phobia and obsessive disorders observed in adolescents than in children. Child presented more fear of being hurt or separation anxiety.

As stated by Ghosh and collaborators in their work on the psychosocial impact of COVID-19 [21], quarantine had an immediate and lasting psychosocial impact due to a radical change in the lifestyle of children and adolescents.

A US study published in April 2022 in the Centers for Disease Control and Prevention's Morbidity and Mortality Weekly Report covering the period January to June 2021 and involving 7705 students aged 14 to 18 years shows alarming figures. Indeed, 44.2% of the students surveyed report a persistent feeling of sadness and loss of hope. A proportion of 19.9% had seriously considered suicide and 9.0% had attempted it [22].

Children and adolescents who have been infected with COVID-19 show post-infection sequelae, as noted in a study conducted at the Beijing Children's Hospital with 45% anxiety, 22% insomnia and 13% depressive symptoms [23].

Anxiety and fear induced by the pandemic, social isolation due to confinement, loneliness [24, 25], fear of the loss of a loved one or sadness related to grief [21] and the risk of violence and abuse in a context of family confinement [26] are elements that may have led to a deterioration in the mental health of children and adolescents during the COVID-19 pandemic.

One of the indicators used to measure mental health is the use of psychotropic drugs.

The aim of this study was to assess the changes in psychotropic drug classes dispensed to children and adolescents living in Limousin, a French region, over the period 2018 to 2021.

## Method

The principal endpoint was the number of psychotropic drugs reimbursed using French health insurance reimbursement data for the period of 2018–2021. This study was conducted in the former administrative region of Limousin, consisting of the following three departments: Corrèze, Creuse and Haute-Vienne. In 2020, the latest population census counted 130,457 children and adolescents compared with 665,700 adults [27].

A comparison of weekly averages of patients having had at least one psychotropic drug over the study period (total number and by pharmacological class) was carried out:

- between and within each year,
- between two periods: 2018–2019, the reference period before the pandemic, and 2020–2021, the period affected by the COVID-19 pandemic,
- between the periods of freedom restriction and the periods without restriction within the 2020–2021 pandemic episode.

There are several medical databases in France produced and managed by the national health insurance fund. The choice fell on the database called "Système National des Données de Santé" (SNDS) because it presents the highest level of detail of reimbursements. The data collected in this database make it possible to obtain demographic characteristics of insured persons (date of birth, gender) and information on the nature of medical procedures reimbursed, including the reimbursement of drugs.

Data from all schemes (with the exception of the special schemes of the National Assembly, the Senate and the military) are fed into the SNDS representing 97% of the population [28]. The database used complies with the requirements of the “Commission Nationale Informatique et Liberté” (CNIL), which guarantees the anonymity and security of the data.

A request was made by the regional health insurance medical service to collect data from all minor patients aged 0 to 17 years who had received reimbursement for psychotropic drugs belonging to the Anatomical Therapeutic Chemical (ATC) classification: N05A (antipsychotics), N05B (anxiolytics), N05C (hypnotics and sedatives), N06A (antidepressants) and N06B (psychostimulants) from 2018 to 2021. In France, the drugs in these classes are only available on doctor’s prescription, so the data obtained is almost complete. For each reimbursement, a unique code identifying the beneficiary, the gender, the date of birth of the beneficiary and the date of reimbursement were extracted. Each line on the output table corresponds to the reimbursement of a psychotropic drug pack (box, bottle, etc.). This reimbursement is generated when a dispensing is carried out according to a prescription and when this dispensation is communicated to the health insurance.

Only one exclusion criterion was retained for this study: molecule without indication in psychiatry which are midazolam and caffeine. Furthermore, due to the marketing in March 2020 of the melatonin-based hypnotic indicated for the treatment of insomnia in children and adolescents aged 2 to 18 years with an autism spectrum disorder, the consumption data may be biased. To avoid bias, some general results were presented without the hypnotics.

Data collection and data management were performed using an ad-hoc Excel form (version 16.62). The database was stored in secured servers of the CH Esquirol hospital. Statistical analyses were performed with XLSTAT module (version 2022.2.1.1305).

### Statistics analyses

The number of patients per week with at least one reimbursement for psychotropic drugs (total number and per therapeutic subclass) was extracted from the database. The annual weekly means of minors who received at least one reimbursement for a psychotropic drug and for a psychotropic class were compared by an ANOVA test. In the case of a significant ANOVA, Tukey’s test was used to compare all possible pairs of means.

The comparison of the average weekly number of patients who had at least one psychotropic drug reimbursement over the pre-COVID (2019 to 2019) versus COVID (2020 to 2021) periods was performed. These means were compared using a one-tailed z-test with an alpha significance level of 0.025 because lower consumption is presumed before COVID-19.

Similarly, the comparison of the average weekly number of patients having had at least one psychotropic drug reimbursement over the periods of confinement, curfew and absence of restrictions was performed using a two-tailed z-test.

In order to make a comparison by year of the evolution of consumption, we studied the evolution of the demography within each French department studied. We used data from the French National Institute of Statistics and Economic Studies and its estimation tools [29]. We extracted and estimated the number of children and adolescents in 2018, 2019, 2020 and 2021 by age group and by department studied. We calculated the proportion of the total number of children and adolescents having received reimbursement for one of the psychotropic drugs studied in relation to the number of minors in the region studied. The evolution according to the year and the health period is thus described. The proportions were compared using a Chi square test at a significance level of 0.05.

#### Ethical considerations

This work has been approved by an ethics committee registered under no. 66–2023-10.

## Results

A total of 49,932 lines of reimbursements for psychotropic drugs was collected in the database. The psychotropic drugs extracted by ATC classification are detailed exhaustively in the appendix. The analyses were performed on 48,454 lines of reimbursements, after exclusion of midazolam and caffeine prescription.

A total of 7949 patients aged 0 to 18 years was studied (Table 1). The average age was 12.1 ± 4.7 years and the sex ratio was 0.97 (M/F).

**Table 1:** Demographic characteristics of the study population by year

Number of patients	Total 2018 to 2021 (n = 7949)	2018 (n = 2595)	2019 (n = 2696)	2020 (n = 2759)	2021 (n = 3274)	P-value^(a)^
Age						
Mean (years)	12.1	11.9	12.0	12.2	12.2	0.0199
SD	4.7	4.5	4.5	4.3	4.4	
Sex						
Male	3917 (49.3%)	1409 (54.3%)	1486 (55.1%)	1445 (52.4%)	1680 (51.3%)	/
Female	4032 (50.7%)	1186 (45.7%)	1210 (44.9%)	1314 (47.6%)	1594 (48.7%)	

^(a)^ANOVA test, alpha threshold of 0.05, if p < alpha: differences between some means are statistically significant

**Fig. 1:**
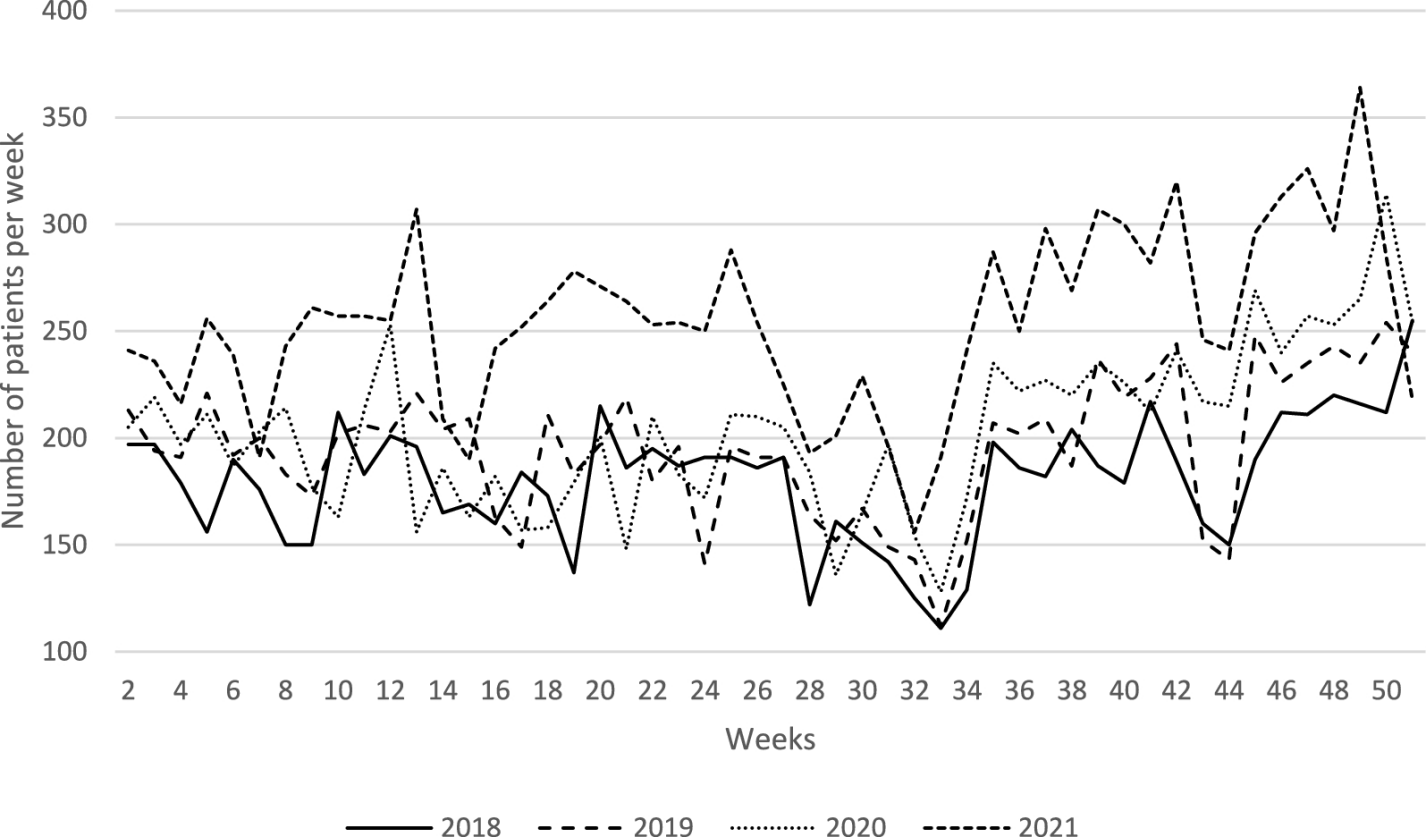
Number of patients who received at least one psychotropic drug reimbursement per week and year

Figure 1 shows the number of patients receiving reimbursement for at least one psychotropic drug per week over the four years of the study. The graph shows a sharp break in the curve for the year 2021.

The number of reimbursed lines and the number of patients is showed for each psychotropic drug class (Table 2).

**Table 2:** Number of lines and patients with psychotropic drug reimbursement per year and therapeutic class

	Antipsychotics	Anxiolytics	Sedatives and hypnotics	Antidepressants	Psychostimulants
Number of lines reimbursed (difference n–1%)					
2018	2803	2965	48	1411	2762
2019	3135 (+ 11.8%)	2996 (+ 1.0%)	82 (+ 70.8%)	1533 (+ 8.6%)	3140 (+ 13.7%)
2020	3126 (– 0.3%)	3201 (+ 6.8%)	469 (+ 472.0%)	1821 (+ 18.8%)	2891 (– 7.9%)
2021	3307 (+ 5.8%)	3701 (+ 15.6%)	1479 (+ 215.4%)	2242 (+ 23.1%)	3367 (+ 16.5%)
Line reimbursed gap between 2018 and 2021 (%)	504 (+ 18.0%)	736 (+ 24.8%)	1431 (+ 281.1%)	831 (+ 58.9%)	605 (+ 21.9%)
Number of patients (difference n–1%)					
2018	415	1696	38	404	419
2019	465 (+ 12.0%)	1742 (+ 2.7%)	45 (+ 18.4%)	402 (– 0.5%)	477 (+ 13.8%)
2020	467 (+ 0.4%)	1726 (-0.9%)	147 (+ 226.7%)	423 (+ 5.2%)	472 (– 1.0%)
2021	512 (+ 9.6%)	2047 (+ 18.6%)	300 (+ 104.1%)	541 (+ 27.9%)	528 (+ 11.9%)
Patient gap between 2018 and 2021 (%)	97 (+ 23.4%)	351 (+ 20.7%)	262 (+ 689%)	137 (+ 33.9%)	109 (+ 26.0%)

For antipsychotics, a statistically significant increase in the number of reimbursed lines was observed between 2018 and 2021 (p < 0.0001, Table 3). Concerning the anxiolytics class, we observed a growing increase in the number of reimbursed lines to reach a difference between 2020–2021 of + 15.6% and between 2018–2021 of + 24.8%, which are statistically significant (p < 0.0001, Table 3). Sedatives and hypnotics drugs significantly increased in terms of number of lines and patients between 2018–2021, with + 281.1% and + 689% respectively (p < 0.0001, Table 3). There was also a significant increase in the number of lines reimbursed for antidepressants from 2020, and an increase of + 58.9% was observed between 2018–2021 (p < 0.0001, Table 3). For psychostimulants, there was an increase of + 21.9% between 2018–2021 (p < 0.0001, Table 3). Comparisons of annual averages of the number of patients reimbursed for at least one psychotropic drug in each drug class are summarised in Table 3.

**Table 3:** Means of patient per therapeutic classes, pre-covid/covid comparison and interannual comparison

Drug classes	Antipsychotics	Anxiolytics	Hypnotics and sedatives	Antidepressants	Psychostimulants
Mean (number of patients per week^(a)^) ± SD					
2018	44.7 ± 7.9	55.6 ± 12.5	1.0 ± 0.9	25.8 ± 6.0	53.5 ± 10.83
2019	48.8 ± 8.7	56.5 ± 13.7	1.5 ± 1.1	27.9 ± 6.3	60.8 ± 12.61
2020	48.6 ± 8.4	59.0 ± 14.7	8.3 ± 6.7	32.7 ± 8.7	55.4 ± 12.33
2021	52.3 ± 9.4	69.5 ± 14.5	26.6 ± 7.4	41.7 ± 9.6	65.1 ± 13.88
Comparison between number of patients pre-covid period and covid period					
Number of patients in the years 2018–2019 pre-covid (mean ± SD)	46.7 ± 6.8	56.1 ± 11.9	1.3 ± 0.72	26.8 ± 4.9	57.2 ± 10.6
Number of patients in the years 2020–2021 covid (mean ± SD)	50.5 ± 7.0	64.2 ± 12.1	17.5 ± 6.2	37.2 ± 8.0	60.3 ± 11.0
P-value	0.003	0.0003	< 0.0001	< 0.0001	0.076
Overall inter-annual comparison^(b)^					
F(between groups df, within groups df) = [F-value]	F(3,196) = [6.53]	F(3,196) = [10.59]	F(3,196) = [281.82]	F(3,196) = [41.13]	F(3,196) = [8.97]
P-value	0.0003	< 0.0001	< 0.0001	< 0.0001	< 0.0001
Pairwise inter-annual comparison^(c)^					
2018 vs 2019	0.088	0.990	0.939	0.512	0.019
2018 vs 2020	0.106	0.624	< 0.0001	< 0.0001	0.868
2018 vs 2021	< 0.0001	< 0.0001	< 0.0001	< 0.0001	< 0.0001
2019 vs 2020	1.000	0.808	< 0.0001	0.014	0.134
2019 vs 2021	0.169	< 0.0001	< 0.0001	< 0.0001	0.318
2020 vs 2021	0.143	0.001	< 0.0001	< 0.0001	0.001

^(a)^Average number of patients per week receiving reimbursement for at least one psychotropic drug

^(b)^ANOVA test, alpha threshold of 0.05, if p < alpha: differences between some means are statistically significant

^(c)^Tukey test, alpha threshold of 0.05, if p < alpha: differences between some means are statistically significant

A comparison by pre-COVID versus COVID period was made. For all categories except psychostimulants, the average reimbursement in the COVID period was significantly higher than the average in the pre-pandemic period (p < 0.0001, Table 4).

**Table 4:** Comparison between average number of patients pre-covid period and covid period and between different periods of restriction

Drug classes	Psychotropic drugs	Psychotropic drugs without hypnotics
Number of patients in the years 2018–2019 pre-covid (Mean ± SD)	188.0 ± 28.0	186.8 ± 28.3
Number of patients in the years 2020–2021 covid (Mean ± SD)	229.6 ± 34.9	212.2 ± 30.8
**P-value pre-covid/covid**	** < 0.0001**	** < 0.0001**
Number of patients in containment (Mean ± SD)	220.5 ± 47.8	207.3 ± 40.6
Number of patients in curfew (Mean ± SD)	248.0 ± 24.3	226.3 ± 20.8
Number of patients without no restriction (Mean ± SD)	225.7 ± 51.7	208.4 ± 42.4
**P-value containment/curfew**	**0.023**	0.061
**P-value containment/no restriction**	0.699	0.916
**P-value curfew/no restriction**	0.056	0.063

**Fig. 2:**
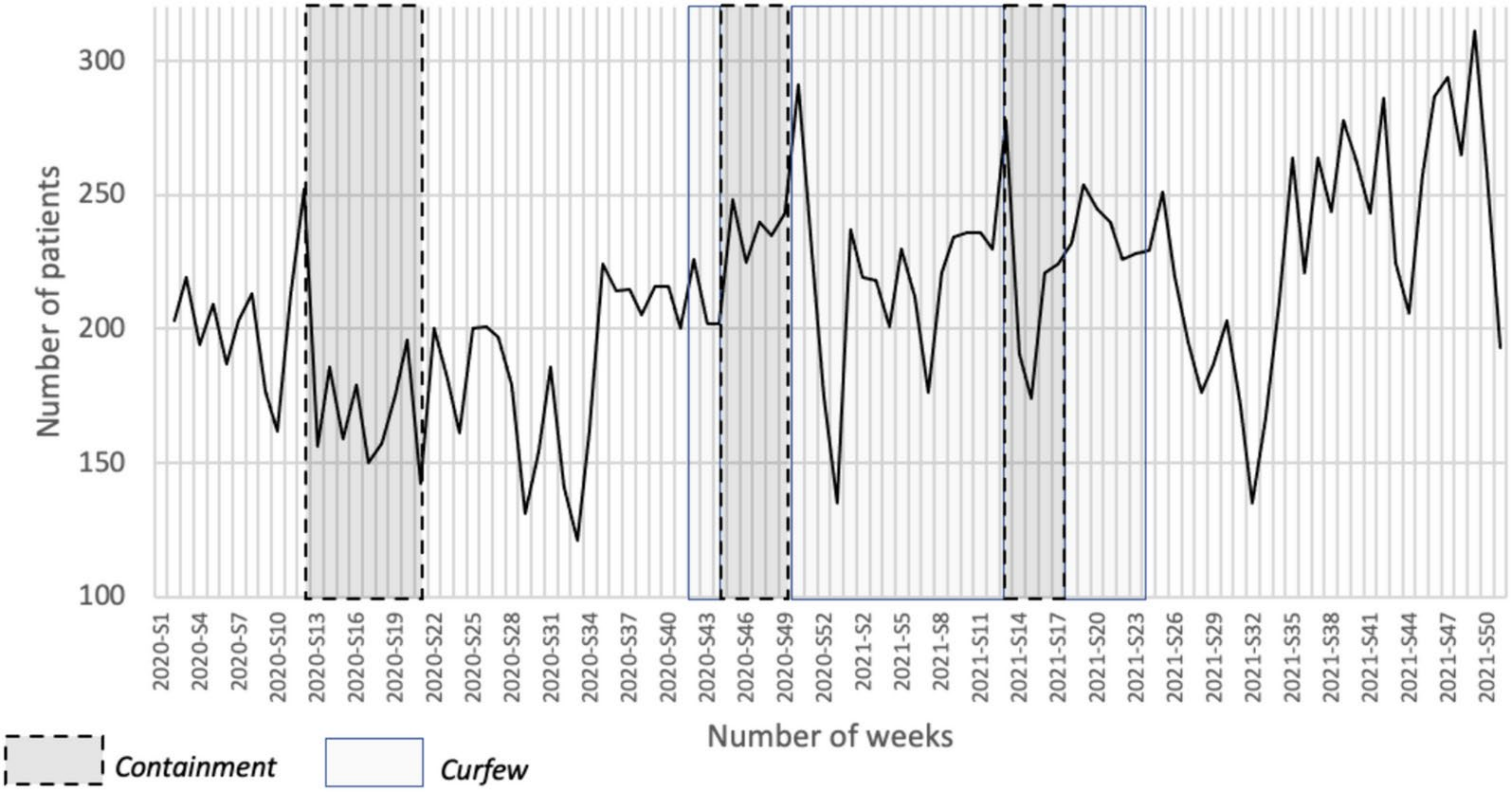
Period of restriction on psychotropic drugs reimbursements by week

Figure 2 shows the evolution of the number of patients receiving at least one psychotropic drug per week in 2020 and 2021, overlaid on the containment and curfew periods. Table 4 shows the comparison of the average number of patients between the lockdown, curfew and non-restriction periods for all psychotropic drugs and all psychotropic drugs except hypnotics. An increased between lockdown and curfew periods was found (p-value 0.023) for all psychotropic medicines, with the other comparisons not being statistically different.

The prevalence of patients receiving reimbursement for at least one psychotropic drug increased to 1.7% to 2.0% between 2018 and 2021 (p < 0.0001). The result is similar when the hypnotics class is removed (1.7% to 1.8%, p = 0.004).

## Discussion

The aim of this study was to determine the short-term impact of COVID-19 on use of psychotropic drugs in the pediatric population. The main finding was a significant increase in dispensing of psychotropics in the years affected by the COVID-19 pandemic, with a particularly strong increase in 2021. This observed increase in dispensing is likely to be correlated with a deterioration in the mental health of young people by the pandemic context as highlighted in a Finnish study which reports an increase in the incidence of primary care from 151.7 per 1000 adolescents in 2019 to 193.6 in 2020 and 306.7 in 2021 [30]. Again, the greatest impact is observed during 2021.

The analysis by therapeutic class shows.

For antipsychotics, another studies have reported an increase in the prevalence of patient using antipsychotics [5, 30]. An increase in the dispensation of antipsychotics may suggest an increase in the pathologies treated with these drugs, such as psychotic, thymic or behavioral disorders. The stability of consumption between 2019 and 2020 means that the effect of the health crisis is not immediate but rather distant.

Concerning anxiolytics, 351 additional youth patients received a reimbursement for this class of psychotropic drugs between 2018 and 2021. A study highlighted an increase of anxiety disorder with + 2% to + 20% of new diagnoses [31] depending on age. Other research has shown up to 39% of anxiety during lockdown in 2020 [32] and 25,7% in 2021 [33] versus 14,9% pre-pandemic anxiety level [34]. On the contrary, some studies find an important decrease in immediate reaction to the restrictions [35, 36] and put forward as hypotheses the closure of schools during this period and the reduction in medical consultations. However, we found no difference directly linked to the restrictions (Table 4). An increase in the number of primary care visits for anxiety diagnoses is observed in Finland with a difference 2019 vs 2020 of + 39% and 2019 vs 2021 of + 128%. An another French study found a high increase in the number of patients consuming anxiolytics (p-value = 0.02) [37]. All these works, including our study, confirms that the health crisis is one of the factors led to a significant increase in use of anxiolytics among youth.

The hypnotics and sedatives class is characterized by a very marked increase in dispensing from March 2020. These results are difficult to interpret because several phenomena may be associated: 1- new reimbursed melatonin-based medicine in France from March 2020, 2- non-reimbursed hypnotics (over the counter) not studied because not included in reimbursements, and 3- some anxiolytics (benzodiazepines), antipsychotics (cyamémazine) and antihistamines (alimemazine) prescribed for sleep disorders. Our hypothesis for this increase is the possible shift of off-label prescriptions to the new reimbursed melatonin-based drug or a greater need among young people. One study highlighted an increase in the number of primary care visits for sleep disorders compared to 2019 of + 79% in 2020 and + 224% in 2021 but found a parallel decrease in the use of hypnotics [30].

The depression generated among young people in connection with the COVID-19 has been widely suspected. Results from reimbursed data for this drug class among youth confirm an increase in dispensing between 2018 and 2021 (+ 58.9% reimbursed lines, + 33.9% patients, Table 2). Indeed, the number of new diagnoses of depression have increased by + 2% to + 32% depending on age according to a German study [31]. The same is true in Finland, with a higher number of consultations in town for depressive episodes, associated with an increase in the use of antidepressants [30]. A Finnish study found an increase in the use of antidepressants among 6–12 year old school children of + 14% in the year 2021 [38]. It should be remembered here that the prescription of an antidepressant is indicated for moderate to severe depressive syndromes but also used for generalized anxiety disorder. In view of all these results, the hypothesis that the context of the pandemic is at the origin of new diagnoses or of the aggravation of pre-existing depression or anxiety disorders (psychotherapy alone to combined with pharmacological treatment).

Contrasting results were observed between years for psychostimulants. A decrease of almost half in the incidence of psychostimulants was observed over the second quarter of 2020 in Canada [35]. A decrease of dispensing was observed in 2020 which we presume is related to the school’s closure for several weeks before the implementation of distance learning courses. Indeed, 16% of parents of children with ADHD said in an Australian survey that treatment is not necessary during school closures [39]. When the data were analyzed globally, i.e. comparing 2018–2019 versus 2020–2021, the difference was not significant (Table 3).

In general, a low period, with a decrease in the number of patients having had a reimbursement, is noted whatever the year in the periods correspond to the summer and autumn school holidays. This observation can be linked to a frequent practice in child psychiatry which consists of limiting the prescription of certain psychotropic drugs such as psychostimulants by reserving them for the school periods and to a lesser need for psychotropic drugs such as anxiolytics outside the school context.

When the impact of the health crisis was assessed by comparing the two periods 2018–2019 and 2020–2021 for the reimbursement of psychotropic drugs and for each drug class, it appeared that the average weekly dispensation of the pre-pandemic years was significantly lower than the average of the pandemic years. All classes of psychotropic drugs were increased except psychostimulants. These results show, in particular, the increase in the use of antidepressants and anxiolytics among youth in the ex-Limousin region during the COVID-19 pandemic.

Apart from the health crisis caused by the COVID-19 pandemic, there is a natural trend towards an increase in psychotropic drug prescriptions among children and adolescent, a process that has been underway for a decade. Between 2014 and 2018, among 0–20 year-olds in France, we observed an increase of + 17.0% for antipsychotics, + 14.0% for antidepressants and normothymics, and + 40% for psychostimulants. For hypnotics and anxiolytics, the trend was for a decrease of– 4.9% [40].

The results in Table 4 show that there is no direct increase because of the restrictions. The significant difference found for all the psychotropic drugs between the confinements and the curfews is certainly linked to the progressive increase in prescriptions of the new drug of melatonin. Indeed, we interpret it as this difference is no longer significant when the hypnotics class is withdrawn.

The progression of the prevalence of psychotropic drugs is significant when the pre-pandemic years were compared to the pandemic years and highlight a real increase in the number of reimbursements for psychotropic drugs in youth.

The impact of the COVID-19 crisis is assumed to be multi-factorial, with examples including the anxiety-provoking media presentation of the pandemic context [13], an imbalance in the parent–child relationship [13, 41, 42], social isolation [43, 44], fear of loss of a loved one/bereavement [13, 21] or intra-family violence/abuse [21, 26].

### Limitations

It is important to note that reimbursement data is derived from a medical prescription, but the fact that a medicine is reimbursed does not necessarily mean that it will be taken by the youth. Medication use is an indicator that we assume correlates with associated mental disorders, so this shortcut may be a source of bias.

The data provided by the health insurance is only considered complete 6 months after the date of care. Therefore, the results presented, when they include the year 2021, may be underestimated.

This study excludes the use of non-reimbursed drugs such as doxylamine indicated for the treatment of insomnia or self-medication. It also excludes psychotropic drugs found in the family pharmacy which can be given to minors without a prescription [45].

Finally, the limitations of the ATC classification must be borne in mind. Some drug has psychiatric indication like alimemazine, an antihistamine (R06) for insomnia, but non classified as a psychotropic drug and non-included in this study. Similarly, cyamemazine may be prescribed for its sedative properties to help people fall asleep, even though it is classified as an antipsychotic drug according to its ATC code. Same for benzodiazepines such as clonazepam which are indicated as anticonvulsants.

## Conclusion

This study highlighted a significant increase in the reimbursement of psychotropic drugs among children and adolescents in the French region of Limousin, which may reflect a deterioration in their mental health. The increase in psychotropic drug use was continuous and progressive throughout the pandemic, particularly for anxiolytics and antidepressants. Future studies should continue to monitor psychotropic drug use in this population, to detect the long-term impact of the pandemic.

## Supplementary Information

Supplementary material 1

## Data Availability

No datasets were generated or analysed during the current study.
