# Supplementary material for: Impact of the COVID-19 health crisis on psychotropic drug use in children and adolescents in France
Source: Child Adolesc Psychiatry Ment Health. 2024 Sep 16;18:119. doi: 10.1186/s13034-024-00806-z (PMC11406773; doi:10.1186/s13034-024-00806-z)
Supplement: Supplementary file 1 — Supplementary material 1 [file 13034_2024_806_MOESM1_ESM.docx]

APPENDIX: List exhaustive of ATC codes extract from reimbursement of psychotropic drugs for children and adolescents.

| **ANTIPSYCHOTICS** | |  | **HYPNOTICS & SEDATIVES** | |
| --- | --- | --- | --- | --- |
| N05AA01 | CHLORPROMAZINE |  | N05C | HYPNOTICS AND SEDATIVES (PHYTOTHERAPY) |
| N05AA02 | LEVOMEPROMAZINE |  | N05CD06 | LORMETAZEPAM |
| N05AA06 | CYAMEMAZINE |  | N05CD08 | MIDAZOLAM |
| N05AC01 | PERICIAZINE |  | N05CD11 | LOPRAZOLAM |
| N05AD01 | HALOPERIDOL |  | N05CF01 | ZOPICLONE |
| N05AD05 | PIPAMPERONE |  | N05CF02 | ZOLPIDEM |
| N05AF05 | ZUCLOPENTHIXOL |  | N05CH01 | MELATONIN |
| N05AH01 | LOXAPINE |  | N05CM09 | VALERIAN |
| N05AH03 | OLANZAPINE |  | N05CM11 | BROMIDES |
| N05AH04 | QUETIAPINE |  | **ANTIDEPRESSANTS** | |
| N05AL01 | SULPIRIDE |  | N06AA02 | IMIPRAMINE |
| N05AL03 | TIAPRIDE |  | N06AA04 | CLOMIPRAMINE |
| N05AL05 | AMISULPRIDE |  | N06AA06 | TRIMIPRAMINE |
| N05AN01 | LITHIUM |  | N06AA09 | AMITRIPTYLINE |
| N05AX08 | RISPERIDONE |  | N06AA16 | DOSULEPINE |
| N05AX12 | ARIPIPRAZOLE |  | N06AB03 | FLUOXETINE |
| N05AX13 | PALIPERIDONE |  | N06AB04 | CITALOPRAM |
| **ANXIOLYTICS** | |  | N06AB05 | PAROXETINE |
| N05BA01 | DIAZEPAM |  | N06AB06 | SERTRALINE |
| N05BA04 | OXAZEPAM |  | N06AB08 | FLUVOXAMINE |
| N05BA05 | POTASSIUM CLORAZEPATE |  | N06AB10 | ESCITALOPRAM |
| N05BA06 | LORAZEPAM |  | N06AX03 | MIANSERINE |
| N05BA08 | BROMAZEPAM |  | N06AX11 | MIRTAZAPINE |
| N05BA09 | CLOBAZAM |  | N06AX14 | TIANEPTINE |
| N05BA11 | PRAZEPAM |  | N06AX16 | VENLAFAXINE |
| N05BA12 | ALPRAZOLAM |  | N06AX17 | MILNACIPRAN |
| N05BA16 | NORDAZEPAM |  | N06AX21 | DULOXETINE |
| N05BA18 | ETHYLE LOFLAZEPATE |  | N06AX26 | VORTIOXETINE |
| N05BA21 | CLOTIAZEPAM |  | **PSYCHOSTIMULANTS** | |
| N05BB01 | HYDROXYZINE |  | N06BA04 | METHYLPHENIDATE |
| N05BE01 | BUSPIRONE |  | N06BA07 | MODAFINIL |
| N05BX03 | ETIFOXINE |  | N06BC01 | CAFFEINE |
|  |  |  | N06BX03 | PIRACETAM |
